# Editorial: Left atrial appendage occlusion: basic and clinical

**DOI:** 10.3389/fcvm.2023.1325397

**Published:** 2023-11-15

**Authors:** Nan Zhang, Wilber Su, Yuxin Li, Junxian Song, Tong Liu, Ruiqin Xie

**Affiliations:** ^1^ Tianjin Key Laboratory of Ionic-Molecular Function of Cardiovascular Disease, Department of Cardiology, Tianjin Institute of Cardiology, Second Hospital of Tianjin Medical University, Tianjin, China; ^2^ Banner - University Medical Center Phoenix, Banner Health, Phoenix, AZ, United States; ^3^ Division of Cell Regeneration and Transplantation, Nihon University School of Medicine, Tokyo, Japan; ^4^ Department of Cardiology, Peking University People’s Hospital, Beijing, China; ^5^ Department of Cardiology, the Second Hospital of Hebei Medical University, Shijiazhuang, China

**Keywords:** atrial fibrillation, left atrial appendage occlusion, stroke, prevention, radiofrequency ablation

**Editorial on the Research Topic** Left atrial appendage occlusion: basic and clinical

Atrial fibrillation (AF) represents the most common sustained cardiac arrhythmia worldwide, with an estimated prevalence of 2%–4% in adults, which is associated with substantial morbidity and mortality (1). Therefore, AF poses significant burden to patients, physicians, and healthcare systems globally. Overall, AF is associated with a 5-fold increased risk of ischemic stroke, and accounts for nearly 25% of the cerebrovascular accidents annually in the United States (2). Avoiding stroke therapy represents one of the vital elements of the simple Atrial fibrillation Better Care (ABC) holistic pathway, including systemic anticoagulation and nonpharmacologic strategies (1). Anticoagulant treatment is the first-line treatment for AF thromboprophylaxis, whereas anticoagulant-related bleeding complications and nonadherence are barriers to effective anticoagulation. Previous study showed that approximately 90% of thrombi in AF patients originate from the left atrial appendage (3). Therefore, left atrial appendage occlusion (LAAO) has become the main alternative for stroke prevention in nonvalvular AF patients with high bleeding risk, with a reported implantation success rate up to 98% (4). Increasing operator experience, evolving device technology, and upcoming trials of new LAAO devices are reassuring aspects of LAAO future (5). LAAO has currently entered the rapid progress era of independent innovation and continuous technology improvement, including the combination of catheter ablation procedures. However, it also brings some efficacy and safety issues that need to be further addressed. This Research Topic on *Left Atrial Appendage Occlusion: Basic and Clinical* presents some of the latest ideas, arguments, and evidence from the investigations into LAAO, which shed light on the best guidance methods for LAAO.

Catheter ablation is a well-established treatment for maintenance of sinus rhythm and symptom improvement among patients with AF, whereas its role in long-term stroke prevention remains unproven. According to the 2020 European Society of Cardiology (ESC) guideline, long-term continuation of systemic anticoagulation beyond two months post ablation is based on the patient's stroke risk profile (1). Since both the catheter ablation and LAAO require percutaneous catheter instrumentation of the left atrium, therefore, a subset of patients may benefit from concomitant intervention of catheter ablation and LAAO as a two-pronged strategy for rhythm control and stroke prevention, which referred to the one-stop procedure (6). In this research topic, two literatures focused on the safety and efficacy outcomes of the combination of catheter ablation and LAAO. Zhu et al. have compared the outcome of AF patients underwent LAAO with catheter ablation and those without, detected by peri-procedural transesophageal echocardiography. The authors observed a higher risk of residual leak and a smaller device compression ratio in patients with combined approach, in relation to those with LAAO alone, which suggests that residual leak and smaller device compression may be partly associated with catheter ablation, and a larger size of device should be considered to maintain the compression ratio for patients whose release site is closer to the ridge. In another study conducted by Ma et al., the authors have compared the safety and efficacy outcomes between the combination of LAAO with cryoballoon ablation and the combination with radiofrequency ablation among patients with AF. A total of 112 patients have been enrolled in this study. During a one-year follow up, this study found that the risk of peri-device leak, peri-procedural and follow-up adverse events were comparable between these two groups, whereas the procedure time was significantly lower in patients underwent LAAO with cryoballoon ablation, compared to those received LAAO with radiofrequency ablation. Taken together, these two studies may provide some novel insights into the clinical practice and scientific researches of the combined procedure of AF ablation and LAAO. Large-scale and long-term follow-up studies are warranted to verify these findings.

Electrical cardioversion terminates AF in over 90% of cases and is the treatment of choice in haemodynamically compromised patients (7). In a subset of patients with contraindications for catheter ablation or those experienced AF recurrence after prior ablation, direct current cardioversion (DCCV) is an alternate, nonpharmacological choice to restore sinus rhythm and relieve clinical symptoms. Meng et al. have compared the outcomes between AF patients underwent DCCV at the time of LAAO and those underwent LAAO alone. They found that DCCV at the time of LAAO is feasible and safe, with a success rate of 70% and a recurrence rate of 40% at the 3-month follow-up. Compared with LAAO alone, DCCV with LAAO does not increase post-procedural adverse events and is more likely to succeed in patients with younger age and lower HAS-BLED scores, which may provide some guidance for management of patients with contraindications for catheter ablation or AF recurrence after previous catheter ablation, but also require further studies to testify the robustness of these findings.

It has been reported that LAAO has an acceptable procedure-related complication rate of 4% at 30 days post-procedure. Nevertheless, the implantation procedure can cause serious complications, with higher event rates in real-world analyses compared with industry-sponsored studies (1). Pericardial effusion is one of the uncommon but serious complication post-LAAO. Yu et al. have retrospectively included 133 patients with nonvalvular AF undergoing LAAO using the LAmbre device, and observed an incidence of 2.3% for both acute and delayed pericardial effusion. This study suggests that both acute and delayed pericardial effusion are uncommon in patients with AF after LAmbre device implantation, in addition, congestive heart failure and a larger LAA orifice have the potential to serve as independent predictors for the occurrence of pericardial effusion.

In summary, the articles from this Research Topic provide latest advances and novel insights into the efficacy and safety outcomes for LAAO and related procedures, which could provide important implications and guidance for clinical practice in the management of patients with AF. We hope that this Research Topic will stimulate novel ideas, experiments, and further investigations in this research field.

## References

[1] Hindricks G Potpara T Dagres N Arbelo E Bax JJ Blomström-Lundqvist C 2020eEsc guidelines for the diagnosis and management of atrial fibrillation developed in collaboration with the European association for cardio-thoracic surgery (eacts): the task force for the diagnosis and management of atrial fibrillation of the European society of cardiology (esc) developed with the special contribution of the European heart rhythm association (ehra) of the esc. Eur Heart J. (2021) 42:373–498. 10.1093/eurheartj/ehaa612 32860505

[2] Virani SS Alonso A Aparicio HJ Benjamin EJ Bittencourt MS Callaway CW Heart disease and stroke statistics-2021 update: a report from the American Heart Association. Circulation. (2021) 143:e254–743. 10.1161/cir.0000000000000950 33501848 PMC13036842

[3] Aberg H. Atrial fibrillation. I. A study of atrial thrombosis and systemic embolism in a necropsy material. Acta Med Scand. (1969) 185:373–9. 10.1111/j.0954-6820.1969.tb07351.x 5808636

[4] Boersma LV Ince H Kische S Pokushalov E Schmitz T Schmidt B Efficacy and safety of left atrial appendage closure with watchman in patients with or without contraindication to oral anticoagulation: 1-year follow-up outcome data of the EWOLUTION trial. Heart Rhythm. (2017) 14:1302–8. 10.1016/j.hrthm.2017.05.038 28577840

[5] Collado FMS Lama von Buchwald CM Anderson CK Madan N Suradi HS Huang HD Left atrial appendage occlusion for stroke prevention in nonvalvular atrial fibrillation. J Am Heart Assoc. (2021) 10:e022274. 10.1161/jaha.121.022274 34668395 PMC8751840

[6] Phillips KP Walker DT Humphries JA. Combined catheter ablation for atrial fibrillation and watchman® left atrial appendage occlusion procedures: five-year experience. J Arrhythm. (2016) 32:119–26. 10.1016/j.joa.2015.11.001 27092193 PMC4823577

[7] Brandes A Crijns H Rienstra M Kirchhof P Grove EL Pedersen KB Cardioversion of atrial fibrillation and atrial flutter revisited: current evidence and practical guidance for a common procedure. Europace. (2020) 22:1149–61. 10.1093/europace/euaa057 32337542 PMC7399700

